# Development of Meat Substitutes from Filamentous Fungi Cultivated on Residual Water of Tempeh Factories

**DOI:** 10.3390/molecules28030997

**Published:** 2023-01-19

**Authors:** Rachma Wikandari, Daniel Reinhart Tanugraha, Anang Juni Yastanto, Manikharda, Rebecca Gmoser, José António Teixeira

**Affiliations:** 1 Department of Food and Agricultural Product Technology, Faculty of Agricultural Technology, Gadjah Mada University, Yogyakarta 55281, Indonesia; 2 Swedish Centre for Resource Recovery, University of Borås, 50190 Borås, Sweden; 3 Centro de Engenharia Biológica (CEB), Universidade do Minho, Campus de Gualtar, Gualtar, 4710-057 Braga, Portugal; 4 LABBELS—Associate Laboratory in Biotechnology, Bioengineering and Electromechanical Systems, 4710-057 Braga, Portugal

**Keywords:** mycoprotein, residual water, tempeh factory, meat substitute

## Abstract

In recent years, there has been an increased motivation to reduce meat consumption globally due to environmental and health concerns, which has driven the development of meat substitutes. Filamentous fungal biomass, commonly known as mycoprotein, is a potential meat substitute since it is nutritious and has filaments to mimic meat fibrils. The current study aimed to investigate the potential use of a cheap substrate derived from the food industry, i.e., residual water in a tempeh factory, for mycoprotein production. The type of residual water, nutrient supplementation, optimum conditions for biomass production, and characteristics of the mycoprotein were determined. The results showed that the residual water from the first boiling with yeast extract addition gave the highest mycoprotein content. The optimum growth condition was a pH of 4.5 and agitation of 125 rpm, and it resulted in 7.76 g/L biomass. The mycoprotein contains 19.44% (*w*/*w*) protein with a high crude fiber content of 8.51% (*w*/*w*) and a low fat content of 1.56% (*w*/*w*). In addition, the amino acid and fatty acid contents are dominated by glutamic acid and polyunsaturated fatty acids, which are associated with an umami taste and are considered healthier foods. The current work reveals that the residual boiling water from the tempeh factory can be used to produce high-quality mycoprotein.

## 1. Introduction

The world’s population is expected to increase from 7.7 billion today to 9.7 billion by 2050 [1]. The growing population will generate an increased demand for foods containing certain components, such as animal protein, especially in developing countries [2]. This phenomenon results in concerns about sustainability and food security [3]. On the other hand, animal protein can produce higher levels of greenhouse gases than plant-based protein [4]. In addition, an increase in the demand for animal protein will lead to an increase in land use for growing fodder and the conversion of forests, wetlands, and natural grasslands to agricultural land, which negatively impacts greenhouse gas emissions, biodiversity, and other important aspects of ecosystems [5]. In terms of health, excessive meat consumption is associated with health problems. An intake of 50 g per day of processed meat was reported to be associated with a 16%, 17%, 25%, and 8% higher risk of colorectal, colon, anal, and kidney cell cancer, respectively [6]. Therefore, a more sustainable protein source with high protein quality and low environmental impact is urgently needed.

Mycoprotein produced from fungal biomass is a potential protein source due to its favorable taste, high protein content, and environmental friendliness. Mycoprotein is reported to contain similar proteins to those of eggs [7,8] and a high polyunsaturated fatty acid content (40%), low saturated fatty acid content (11%), and low fat content (3.25%) [9]. Mycoprotein has superior sensory properties compared to other meat alternatives, as shown by the highest scores for acceptability [10]. This result could be due to its fiber bundle content, which mimics meat fibers [11]. In addition, mycoprotein production requires less land and water and releases lower greenhouse emissions than beef production [12]. Mycoprotein has been sold in 17 countries, and the demand is projected to increase in the future [13]. Commercial mycoprotein is produced from the biomass of *Fusarium venenatum* grown on glucose. To reduce the production cost, a cheaper substrate is needed to produce mycoprotein, for example, byproducts of the food industry.

The soybean food industry is growing with the increasing consumption of soybean-based food products. This phenomenon is associated with the rising level of public awareness of the importance of soy consumption for human health. Some processed soy products that are widely consumed are soy milk, soy cheese, tofu, etc. In Indonesia, soybeans are mainly used to make tempeh, a fermented food that has become a staple protein source in Indonesia. There are 81,000 tempeh industries in Indonesia, and the number continues to increase yearly. According to the Indonesian Primary Cooperative of Tofu Tempeh, 12.8 million tons of soybean were used for making tempeh in one year [14]. During tempe processing, 200–300 L of liquid waste is generated from 300 kg of soybean [15,16]. Therefore, it is estimated that the tempeh industry produces 0.85–1.28 million m^3^ per year or 2338–3057 m^3^ per day liquid waste. Part of the liquid waste is used to produce biogas, but most of it is disposed into rivers, causing environmental problems. Due to the high carbon and nutrient content of tempeh wastewater [17], this byproduct can be a substrate for producing mycoproteins. The liquid waste in the tempeh industry includes residual waters from soaking, washing, and boiling. The residual process water that has not entered the waste disposal system can be accommodated and not categorized as waste. However, no studies have reported mycoprotein production from residual water from tempeh factories. Therefore, this study investigated the potential use of residual water from a tempeh factory for mycoprotein production. In this study, a filamentous fungus commonly used for making tempeh, *Rhizopus oligosporus,* was used for mycoprotein production. This fungus is generally recognized as safe (GRAS), and its taste has been accepted by consumers for many years. The effects of the type of residual water, nutrient supplementation, and growth conditions on mycoprotein production were studied. Nutritional quality characteristics, including protein content, digestibility, and amino acid and fatty acid profiles, were also determined. The current work is a preliminary study on the suitability of the use of residual water from tempeh factories as a basal medium for mycoprotein production. The conversion of residual water from food processing into mycoprotein not only contributes to reducing the load of pollutants on the environment but also generates protein alternatives to strengthen food supply security.

## 2. Results and Discussion

### 2.1. Effect of Media Composition on Biomass Production

The utilization of food waste and byproducts for cultivating edible fungi not only contributes to providing sustainable food production but also reduces environmental pollution. Soybean is among the most widely used raw materials to produce various products. In Indonesia, 80% of soybeans are used for making tempeh [14]. During tempeh processing, the soybean is subjected to first soaking, first boiling, second soaking, second boiling, inoculation with fungi, and incubation. Therefore, this process generates water from the first soaking and boiling and from the second soaking and boiling. These waters might have different compositions, which could affect the growth of filamentous fungi. Thus, the first step of this research was to characterize the residual water from tempeh processing.

The results presented in Table 1 show that the reducing sugars and protein of all types of residual waters varied from 0.055 to 0.508 g/L and from 0.41–2.62 g/L, respectively. The highest reducing sugar and protein contents were obtained in the residual water from the first boiling (RB1). The high reducing sugar content of RB1 might come from soluble soybean polysaccharide, a water-soluble fraction extracted from the cell wall material of the cotyledons of soybean [18]. In addition, it has been reported that the main soluble carbohydrates in soybean seeds are sucrose and the raffinose family of oligosaccharides (RFO), raffinose, and stachyose [19]. Since the soluble carbohydrate had been dissolved in the first soaking, the sugar concentration was lower in the second soaking and boiling. In addition, after the first boiling, the soybean was dehulled before the second soaking; hence, the soluble carbohydrates might remain in the soybean hull, resulting in lower sugars in the residual water from the second soaking and boiling (RD2 and RB2).

**Figure 1:**
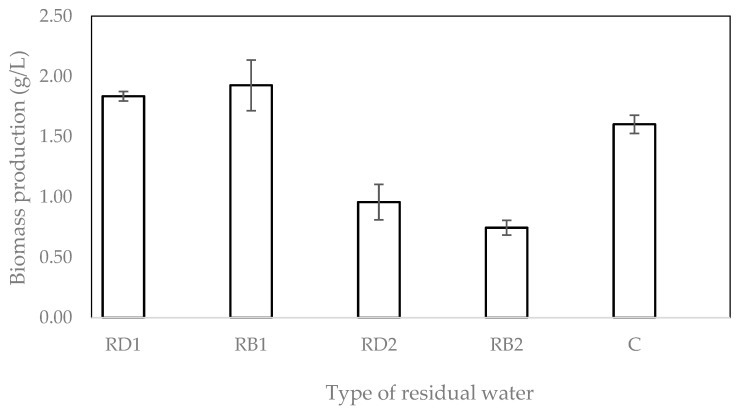
Biomass of *R. oligosporus* in different types of residual waters from tempeh processing.

The biomass production results in Figure 1 show that all types of residual water could be used to cultivate *Rhizopus oligosporus*. In accordance with the reducing sugar and protein contents, the highest biomass production was obtained from RD1, reaching 1.9 g/L.

**Figure 2:**
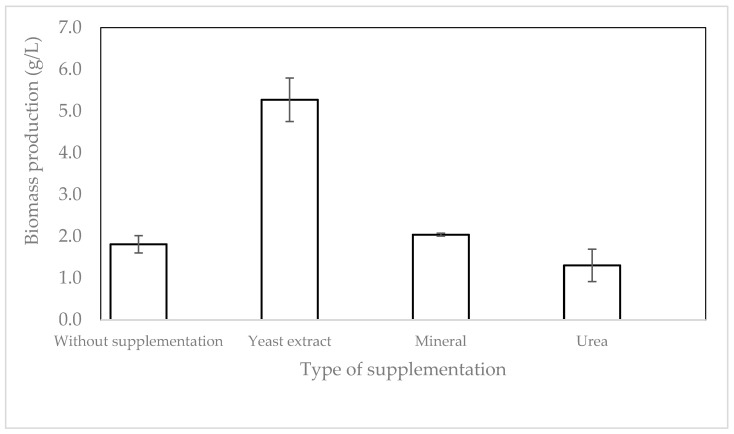
Biomass production from tempeh residual boiling water supplemented with different nutrients.

To evaluate the effect of micronutrients and nitrogen on fungal biomass production, yeast extract, urea, and mineral salts were added to the media. Medium RD 1 without the addition of micronutrients or nitrogen was used as a control. As shown in Figure 2, the addition of yeast extract resulted in the highest fungal biomass, which reached 5.27 g/L (dry basis), increasing the production by two and half times. By adding yeast extract containing 10% nitrogen [20], the C/N ratio was decreased, favoring biomass production [21]. In addition, yeast extract contains 2.5% phosphorus and a broad range of macro- and micronutrients [20], which could provide the nutritional requirement for the growth of filamentous fungi. This result is in accordance with a previous study reporting that the addition of yeast extract at the same concentration as in this study resulted in a two times higher fungal biomass concentration of *Rhizopus arrhizus* 36017 [22]. The addition of yeast extract has also been reported to increase the fungal biomass of *Rhizopus oryzae* by more than five times after 24 h of fermentation [23]. This result reveals that the addition of micronutrient sources is essential for fungal biomass production in tempeh residual water. To reduce the production cost and make the process more sustainable, yeast extract obtained from the residue of the brewing industry could be a potential source to replace the yeast extract used in this study.

### 2.2. Effect of Cultivation Conditions on Biomass Production

**Figure 3:**
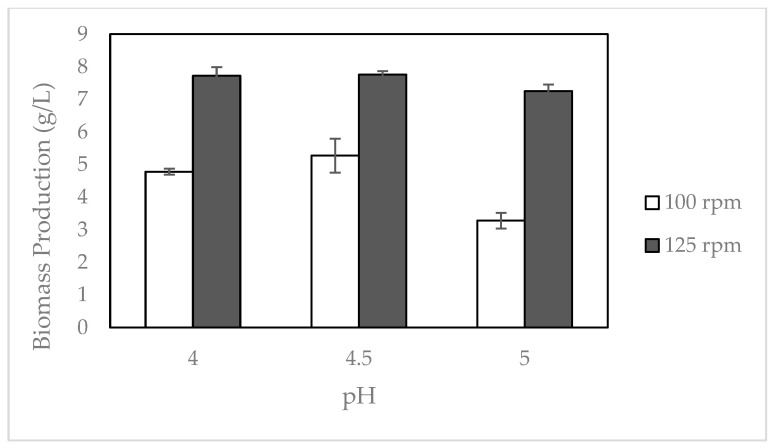
Biomass of *R. oligosporus* cultivated in residual boiling water of tempeh processing under different pH and aeration rates of 100 rpm (□) and 125 rpm (■).

In addition to media composition, environmental factors, such as pH and aeration, might affect fungal biomass production. Therefore, in this study, the fungi were cultivated at pH 4, 4.5, and 5 with agitation at 100 and 125 rpm. The results showed that the highest biomass (7.76 g/L dry basis) was obtained at a pH of 4.5, which agrees with data reported for *Rhizopus stolonifera* [24], and 125 rpm (Figure 3). A higher agitation rate is shown to result in higher fungal biomass. This could be due to better mixing in the culture vessel and better aeration. It has been reported that aeration could increase biomass production by enhancing the mass transfer characteristics with respect to the substrate, product, and oxygen [25].

**Figure 4:**
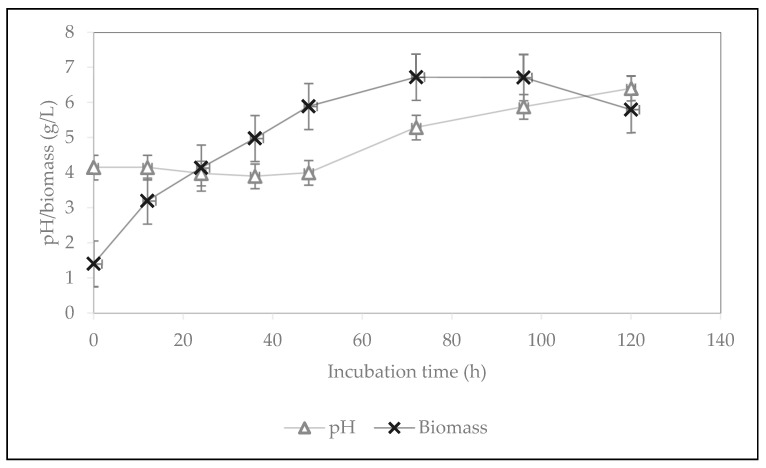
Biomass production and pH changes during cultivation of *Rhizopus oligosporus* on residual water from the first boiling of tempeh processing with the addition of yeast extract and incubation at pH 4.5 and 125 rpm.

The profile of the growth of filamentous fungi and the pH changes under the optimum conditions are presented in Figure 4. Both fungal biomass production and pH increased with an increase in incubation time. The highest fungal biomass was obtained at 72 h. During fermentation, the pH increased from 4 to 6.4. The reason for this could be the presence of proteolytic enzymes produced by *Rhizopus oligosporus*. The enzyme degrades proteins into amino acids and causes an increase in dissolved nitrogen and ammonia content, which raises the pH value. In addition, the increase in pH could be due to the dissolution of water-soluble organic acids produced from complex compounds, such as fats, proteins, and oligosaccharides [26].

### 2.3. Nutritional Quality of Fungal Biomass Cultivated on Residual Water of First Boiling in Tempeh Processing

For further application of fungal biomass as food, the nutritional quality of mycoprotein was evaluated, and the results are presented in Table 2. The fungal biomass contained 19.44% (wet basis) protein. This number is slightly lower than that of beef and higher than that of mycoprotein produced from *Fusarium venenatum*, milk, eggs, soybeans, and wheat [27]. Therefore, it is a potential protein source. To evaluate the protein quality, the protein digestibility of the fungal biomass was determined and was found to be similar to that of tempeh but lower than that of beef and egg. The low digestibility might be related to the presence of the cell wall of filamentous fungi. The cells of filamentous fungi contain glucans (30–80%), chitin and chitosan (1–15%), mannans and galactomannans, and glycoproteins [28,29]. It has been reported that chitin is hard to digest [30], which might be the reason for the poor digestibility of mycoprotein. The digestibility of mycoprotein obtained in this study (45.48%) was lower than that of commercial protein (78%), which might be due to the different types of fungi used for mycoprotein production [31]. In addition, the lower digestibility could be caused by a higher fiber content obtained in this study (8.51%) than commercial mycoprotein (6%) [31]. Fiber is reported to reduce the digestibility of fat and protein [32]. The lipid content was lower than that of beef, chicken, egg, and tempeh. In addition, the lipid content of the fungal biomass obtained in this study (1.56%) was lower than those reported by previous studies, 3–64% [33]. Furthermore, mycoprotein has a high crude fiber content.

The amino acid composition is presented in Table 3. The results show that the fungal biomass contains 16.98 g/100 g of essential amino acids, which is three times higher than that of mycoprotein from *Fusarium venenatum* (4.59 g/100 g). [34]. Essential amino acids are amino acids not synthesized by the human body that, thus, must be ingested from the diet. As seen in Table 3, consumption of 100 g of the fungal biomass could fulfill the requirements of all essential amino acids for a person with a weight of 60 kg, except for methionine, which would require a consumption of 200 g of fungal biomass. The amino acid content of the fungal biomass was lower than that of beef but higher than that of soybean (Table 3). Glutamic acid was the most abundant amino acid. Glutamic acid is responsible for a savory/umami taste. It has been reported that the glutamic acid content of fermented products of *R. oligosporus* strongly correlates with their overall acceptance for human consumption [35]. Hence, there is a possibility that the taste of the fungal biomass generated in this study will be acceptable to consumers.

**Figure 5:**
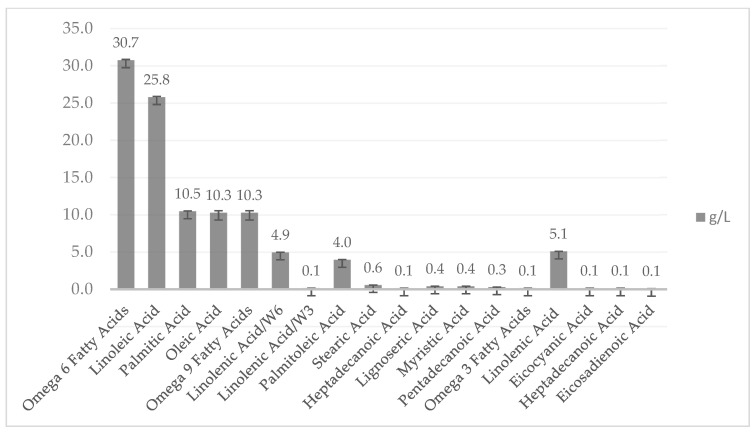
Fatty acid composition of fungal biomass cultivated on residual water from the first boiling of tempeh processing with the addition of yeast extract and incubated at a pH of 4.5 and aeration of 125 rpm.

The fatty acid composition is presented in Figure 5. The results show that the fatty acid composition was dominated by polyunsaturated fatty acids (3.0%), followed by monounsaturated fatty acids (1.5%), and saturated fatty acids (1.2%). The highest fatty acid content was omega-6 (3.0%), followed by linoleic (2.6%), palmitic (1.0%), and omega-9 (1.0%). In general, polyunsaturated fatty acids (PUFAs) are considered to be healthier than other types of fatty acids. PUFAs are reported to lower the risk of cardiovascular disease and play an important role in nervous system development, maintenance, and function.

The high contents of protein, essential amino acids, fiber, and polyunsaturated fatty acids and the low fat content of the fungal biomass cultivated on the residual boiling water of tempeh processing suggest that it is a potential alternative protein source with high nutritional quality. In addition, the conversion of residual waters into nutritious fungal biomass will not require massive equipment installation, as the process uses the same fungi for making tempeh and mycoprotein so that the process can be easily integrated. Furthermore, the tempeh factory could generate extra income from their byproducts. Since the fungi used for mycoprotein production are similar to those used for making tempeh, which has been consumed for centuries, this production method has reduced risk in regard to the safety of the mycoprotein. The type of substrate for growing the fungi might also affect the product’s safety. In the current work, the substrate is derived from tempeh processing, in which no chemical is added throughout the process. In addition, the residual water is taken directly from the soaking basin or boiler before entering the wastewater pipeline; thus, it does not mix with other waste streams in the factory. The use of waste streams obtained without any further processing (just heating) from the tempeh factory is a critical factor in validating biomass production for use as food or feed. Considering that the tempeh production process complies with all the safety issues for a food product, no significant concerns are expected concerning the safety of the biomass obtained in this manner. It may be argued that residual pesticides from soybean might be present in the wastewater used. However, this should not be a concern, once again, due to the quality control applied to the soybeans used in tempeh production. The results of this study reveal that the conversion of food industry byproducts could reduce the load of environmental pollution; hence, mycoprotein produced from food processing residues could be an example of future sustainable food production.

## 3. Materials and Methods

### 3.1. Microorganisms

*Rhizopus* strains were isolated from traditional tempeh starter cultures collected from Yogyakarta Province, Indonesia. The fungal strain was identified as *R. oligosporus* using a molecular method [35]. The isolates were grown in PDA (Merck, Darmstadt, Germany) and kept at 4 °C until use.

### 3.2. Residual Water from Tempeh Processing

During tempeh processing, several types of residual water were generated from different processing stages. To prepare the residual water, the soybean was first soaked in water for 24 h with a soybean-to-water ratio of 1:5. The residual water after the first soaking was coded as RD1. The soaked soybeans were boiled for 30 min with a soybean-to-water ratio of 1:1. The residual water from the first boiling was used and coded as RB1. The next step was dehulling. Dehulled cooked soybean was subjected to a second soaking for 24 h with the same ratio of water-to-soybeans as the first soaking, and the remaining water from the second soaking was used and coded as RD2. Subsequently, boiling was repeated for 30 min with the same ratio of water-to-soybeans as the first boiling. The water remaining from the second boiling was used and coded as RB2. A mixture of all types of residual water, named C, was made by mixing the same volume of each type of residual water. All types of residual waters were sterilized prior to use for cultivating the filamentous fungus *Rhizopus oligosporus*.

### 3.3. Microorganism Cultivation of Filamentous Fungi in Residual Waters

To investigate the effect of the type of residual water on biomass production, the fungi were cultivated on all types of residual waters, including RB1, RD1, RB2, RB2, and C. Ten milliliters of spore solution containing 1 × 10^6^ spores/mL was inoculated on 250-mL cotton-plugged Erlenmeyer flasks containing 100 mL of residual water. The flask was then incubated in a shaker bath at 30 °C and 110 rpm for 48 h [39].

To investigate the effect of medium supplementation, 5 g/L yeast extract and 1 g/L urea were added to the RB1 medium. The mineral solution concentration in the culture medium was 7.5 g/L (NH_4_)_2_SO_4_, 3.5 g/L KH_2_PO_4_, 0.74 g/L MgSO_4_.7H_2_O, and 1.0 g/L CaCl_2_.2H_2_O. Medium without any supplementation was used as a control. One hundred milliliters of medium was inoculated with 10 mL of spore solution containing 1 × 10^6^ spores/mL. Subsequently, the medium was incubated at 30 °C and 110 rpm for 48 h.

To investigate the effect of pH and aeration, the fungi were cultivated at initial pH values of 4, 4.5, and 5 with aeration rates of 100 and 125 rpm. The initial pH of the medium was adjusted using 2 M H_2_SO_4_. The same procedure was used for incubation and growth.

To evaluate the changes in pH and biomass production during cultivation, the fungi were cultivated under optimum conditions, and samples were taken at 0, 12, 24, 36, 48, 72, 96, and 120 h of incubation.

### 3.4. Determination of Residual Water Composition

The reducing sugar content in all residual waters was determined by the dinitrosalicylic acid (DNS) method [40]. The protein content was analyzed using the Kjeldahl method [41].

### 3.5. Determination of Biomass Concentration

The wet biomass mycelium was harvested on a screen after 48 h of cultivation, washed with water three times, and kept at −20 °C until use. The biomass was dried at 105 °C for 24 h to obtain dry biomass.

### 3.6. Proximate Analysis of the Mycoprotein

The protein content was analyzed by the micro-Kjeldahl method, the fat content was analyzed by the Soxhlet method, and the ash content was analyzed by thermogravimetry, while carbohydrate contents were determined by difference method [42].

### 3.7. Determination of Protein Digestibility

The digestibility of mycoprotein was analyzed using a method previously described by [43] with modifications. Mycoprotein (0.2 g) was dissolved in 0.2 M buffer solution (pH 2) and 1 mL of 2% pepsin enzyme, followed by incubation at 37 °C for 2 h. Subsequently, the sample was centrifuged at 3000 rpm for 20 min. Five milliliters of supernatant was added to 5 mL of 20% trichloroacetic acid, followed by incubation for 1.5 h and filtration using Whatman filter paper no. 41. Eventually, the protein content was determined using the micro-Kjeldahl method.

### 3.8. Determination of Fiber Content

The fiber content of the mycoprotein was determined using a method previously reported by [44] with modifications. Fifteen milliliters of the sample was mixed with 15 mL of 96% ethanol and filtered three times. The remaining solids were dried and added to 50 mL of 1.25% H_2_SO_4_. The mixture was heated for 30 min, followed by addition of 50 mL of 3.25% NaOH solution and then heated again for 30 min. Subsequently, the mixture was filtered and weighed and then washed with 1.25% H_2_SO_4_ hot water and 25 mL of 96% ethanol. The precipitate and filter paper were removed and heated in an oven at 105 °C until a constant weight was achieved.

### 3.9. Determination of the Fatty Acid Profile

The fatty acid profile of the sample was evaluated using a Clarus series gas chromatograph (PerkinElmer, Waltham, MA, USA) equipped with a DB-FastFAME column (30 m, 0.25 mm, and 0.25 µm i.d) (Agilent, Santa Clara, CA, USA) and a flame ionization detector (FID) with helium as the carrier gas. As much as 1 µL of the sample was injected in split mode. The injection temperature was 240 °C, and the oven temperature was initially set at 50 °C and progressively increased to 230 °C. The data obtained were then compared to fatty acid standards (C_4_–C_24_).

### 3.10. Determination of the Amino Acid Profile

The amino acid profile of the sample was analyzed according to a previous method with modifications [45]. The analysis was performed using Acquity ultrahigh-pressure liquid chromatography (UPLC) equipped with a photodiode array detector (Waters, Milford, MA, USA). Approximately 0.1 g of the sample was hydrolyzed with 5 mL of 6 M HCl at 110 °C for 22 h. The mixture was then transferred to a 50 mL volumetric flask and filtered with 0.45 µm filter paper. Then, 500 µL of filtrate was added to 40 µm α-aminobutyric acid as an internal standard and 460 µL of double-distilled water. Then, 10 µL of the solution was added to 70 µL of AccQ-Fluor borate buffer (Waters, USA) and 20 µL of reconstituted Fluor reagent (Waters, USA). The mixture was then incubated at 55 °C for 10 min, and 1 µL of the filtrate was injected into the UPLC system.

## 4. Conclusions

Residual boiling water from tempeh factories is a suitable medium for the growth of edible filamentous fungi. The addition of yeast extract enhances biomass production, and the optimum growth conditions are obtained at a pH of 4.5 and an aeration rate of 125 rpm. The fungal biomass obtained in this study shows promise for further development as an alternative protein source with high fiber, low fat, and high polyunsaturated fatty acid and glutamic acid contents. Conversion of residual boiling water from tempeh factories not only contributes to solving environmental problems but also strengthens food supply security by providing an affordable high-quality protein source. However, the enhancement of protein digestibility and product development of the fungal biomass should be subject to further studies.

## Figures and Tables

**Table 1:** Reducing sugars and protein content of different types of residual water from tempeh processing.

Substrate *	Protein (g/L)	Reducing Sugar (g/L)
RD1	1.11	0.461
RB1	2.62	0.508
RD2	0.41	0.08
RB2	0.7	0.099
C	1.19	0.055

* note: RD1 = residual water from the first soaking. RB1 = residual water from the first boiling. RD2 = residual water from the second soaking. RB2 = residual water from the second boiling. C = a mixture of all types of residual water.

**Table 2:** Proximate content of fungal biomass *Rhizopus oligosporus* cultivated at pH four and an agitation speed of 125 rpm on residual water of tempeh processing with supplementation of yeast extract.

Proximate Content	(%)
Protein (wb)	19.44
Protein digestibility	45.48
Lipid (db)	1.56
Crude fiber (db)	8.51

**Table 3:** Amino acid composition of mycoprotein, soybean, and beef and their requirement in the human body.

Amino Acids	Mycoprotein Obtained in This Study (g/100 g)	Amino Acids in Soybean g/100 g [36]	Amino Acids in Beef g/100 g [37]	Requirements mg/kg [38]
L-Phenylalanine	1.95	1.929	3.09	14
L-Valine	2.16	1.734	4.48	13
L-Tryptophan	0.56	0.45	0.934	3.5–6
L-Threonine	2.45	1.382	3.43	9
L-Isoleucine	1.62	1.709	3.84	10
L-Methionine	0.36	0	2.37	13
L-Leucine	2.58	2.841	6.18	14
L-Histidine	2.53	1.151	2.94	8–12
L-Lysine	3.05	2.363	6.66	12
L-Serine	1.77	1.35	3.2	-
L-Glutamic Acid	4.21	5.31	6.89	-
L-Alanine	2.96	1.23	4.22	-
L-Arginine	2.66	1.92	4.79	-
Glycine	2.11	1.3	3.1	-
L-Aspartic Acid	2.99	3.2	3.73	-
L-Tyrosine	1.83	0.96	2.71	-
L-Proline	1.36	1.29	3	-
L-Cysteine	1.66	0.64	1.01	-

## Data Availability

Not applicable.
